# The Effect of Berry Consumption on Oxidative Stress Biomarkers: A Systematic Review of Randomized Controlled Trials in Humans

**DOI:** 10.3390/antiox12071443

**Published:** 2023-07-18

**Authors:** Kim S. Stote, Gracie Burns, Kim Mears, Marva Sweeney, Cynthia Blanton

**Affiliations:** 1 Department of Allied Health Sciences, State University of New York, Empire State University, Saratoga Springs, NY 12866, USA; 2 Department of Biology, University of Prince Edward Island, Charlottetown, PE C1A 4P3, Canada; geburns@upei.ca (G.B.); msweeney@upei.ca (M.S.); 3 Robertson Library, University of Prince Edward Island, Charlottetown, PE C1A 4P3, Canada; kmears@upei.ca; 4 Department of Nutrition and Dietetics, Idaho State University, Pocatello, ID 83201, USA; cynthiablanton@isu.edu

**Keywords:** berries, polyphenols, anthocyanins, oxidative stress, antioxidants

## Abstract

Bioactive compounds in berries may scavenge reactive oxygen and nitrogen species by donating electrons to free radicals, thereby protecting DNA, proteins, and lipids from oxidative damage. Evidence shows that berry consumption has beneficial health effects, though it remains unclear whether berries exert a significant impact on oxidative stress in humans. Thus, we performed a systematic review of randomized controlled trials (RCT) to examine the effects of non-acute (more than a single dose and ≥7 days) berry consumption on biomarkers of oxidative stress. Searches were conducted in PubMed, Cochrane Library, and Scopus; results were imported into Covidence for screening and data extraction. The literature search identified 622 studies that were screened, and 131 full-text studies assessed for eligibility. Ultimately, 28 RCTs met the eligibility criteria. Common biomarkers of oxidative stress (antioxidants, DNA damage, isoprostanes, malondialdehyde, and oxidized LDL) were systematically reviewed, and results were reported narratively. Of the approximate 56 oxidative stress biomarkers evaluated in the 28 RCTs, 32% of the biomarkers were reported to have statistically significant beneficial results and 68% of the biomarkers were reported as having no statistically significant differences. More well-designed and longer-term berry RCTs are needed to evaluate biomarkers of oxidative stress.

## 1. Introduction

Oxidative stress is implicated in the pathogenesis of age-related decline in diseases like Alzheimer’s disease, cardiovascular disease, and various cancers [1,2,3,4]. Interventions that emphasize healthful plant-based, polyphenol-rich diets are promoted for preventing and managing diseases related to oxidative stress [5,6,7]. Plant polyphenols are a varied grouping of complex structures. Polyphenols are phenolic compounds classified as flavonoids or non-flavonoids. Flavonoids are comprised of anthocyanins, flavan-3-ols, flavanones, flavones, flavonols and isoflavones. Non-flavonoids are stilbenoids, lignins and phenolic acids [8]. Berries are among the most potent sources of bioactive compounds such as vitamin C, carotenoids, and polyphenols, including anthocyanins [9,10]. Anthocyanins are polyphenolic pigments that are responsible for the red, blue, and purple colors present in berries [11]. Further, there are six common anthocyanins, which include cyanidin, delphinidin, malvidin, pelargonidin, peonidin, and petunidin. Anthocyanins may consist of up to 60% of the total phenolic compounds in berries. However, the quantity and structure of anthocyanins vary greatly in plant foods due to agriculture and processing [9,12]. Increasing research interest has focused on the health benefits of anthocyanins and/or anthocyanin-rich foods, such as berries [13]. Currently, food-based guidelines for anthocyanin intake are not currently available in North America, South America, or Europe. However, China has previously defined a proposed level of 50 mg per day for anthocyanins [14]. Current public health recommendations to increase dietary intake of fruits including berries are determined by the need for sufficient nutrient intake from foods, though some dietary guidelines also consider the contribution of dietary bioactive compounds such as anthocyanins [15]. Global consumption of anthocyanins is dependent upon the region of the world; the mean intake of anthocyanins ranges from 15 mg per day to 50 mg per day [16]. Finland’s adult population has a higher dietary intake of berries, with a mean intake of anthocyanins of approximately 50 mg per day [17]. The adult population of the United States has a lower intake of berries, consuming about 15 mg per day of anthocyanins [18].

The bioactive compounds in berries scavenge reactive oxygen and nitrogen species by donating electrons to free radicals, thereby protecting DNA, proteins, and lipids from oxidative damage [19,20,21]. A number of systematic and narrative reviews have described the benefits of specific types of berries and polyphenols on health outcomes such as cardiovascular disease, metabolic disorders and cancer [10,13,22,23,24]. It remains unclear whether berries and their extracts, collectively, exert a significant impact on biomarkers of oxidative stress in humans. A body of evidence to support the effects of berries on oxidative stress in humans would inform our understanding of the mechanisms linking berry consumption and health outcomes. The objective of this research was to conduct a systematic review to examine the effects of berry consumption on biomarkers of oxidative stress in adults from randomized controlled trials (RCT) involving non-acute (more than a single dose and ≥7 days) berry/berry extract feeding in the last 10 years.

## 2. Materials and Methods

This review was registered on 1 February 2023 in the PROSPERO registry (https://www.crd.york.ac.uk/prospero/display_record.php?RecordID=393595, accessed on 1 February 2023). Additionally, the review was conducted and reported in accordance with the Preferred Reporting Items for Systematic Reviews and Meta-Analyses (PRISMA) 2020 guidelines [25]. The search was conducted and reported in accordance with the Preferred Reporting Items for Systematic Reviews and Meta-Analyses literature search extension (PRISMA-S) guidelines [26]. Peer review of the search strategies was not completed.

### 2.1. Information Sources

The searches were conducted and exported on 24 February 2023 in the following electronic databases: PubMed, Cochrane Library (via Cochranelibrary.org), and Scopus (Elsevier). Grey literature sources included a clinical trial registry (ClinicalTrials.gov).

### 2.2. Search Strategy

An initial list of search terms was developed by a health sciences librarian in collaboration with all authors. The three concepts explored in this review include (1) berries; (2) oxidative stress; and (3) anthocyanins. The berries concept was developed by brainstorming a list of common berries, and was then expanded through a review of biological species. The authors achieved consensus on including blackcurrant as a berry after some debate. Fruit was also included as a search term since PubMed does not have a subject heading for the term berry. Many relevant indexed articles included fruit as a MeSH term, so it was recommended to include that term in the search strategy. Search terms for the oxidative stress concept were developed through discussion of oxidative stress biomarkers. The anthocyanins concept was developed through discussion and controlled vocabulary exploration. The search strategy was finalized in PubMed and then translated to Cochrane Library and Scopus. The reproducible searches for all databases are available at https://doi.org/10.11571/upei-roblib-data/researchdata:790 (accessed on 23 May 2023). The search identified RCTs published from 1 January 2013 to 24 February 2023.

#### Search Filters

The publication type was limited to RCTs, using a modified version of Cochrane’s sensitivity and precision-maximizing filter for PubMed [27]. The search was limited to human studies by using a common search filter for excluding publications that include animals or animals and humans through the use of the NOT Boolean operator. An English language filter and a date range of 2013 to 2023 were also applied.

### 2.3. Inclusion and Exclusion Criteria

This systematic review included only RCTs examining the effect of chronic berry consumption on oxidative stress biomarkers. Eligible interventions were dietary sources of berries, including foods, extracts, and supplements. Eligible trial populations included participants of all ages, ethnicities, sexes, and health–disease statuses. Trials that evaluated the impact of acute berry consumption (with a single dose) were not considered. Animal studies, in vitro studies, and observational studies were also excluded.

### 2.4. Data Extraction

Literature search results were imported into Covidence, an online systematic review software for deduplication, screening, and data extraction. Abstract and title screening was completed by one reviewer. Full-text review, data extraction, and bias assessment were completed independently by two blinded reviewers. Extraction was completed using Covidence’s Extraction 2 tool, which was fully customizable to the needs of the project. Conflicts were resolved by review and discussion with all review authors.

For each included RCT, the risk of bias was judged as high, low, or unclear using Covidence’s Risk of Bias assessment version 1.0. This tool includes the Cochrane Risk of Bias domains of sequence generation, allocation concealment, blinding of participants and personnel, blinding of outcome assessment, incomplete outcome data, selective outcome reporting, and ‘other issues’ [28]. An overall risk-of-bias judgment (either high risk of bias, low risk of bias, or some concerns) was assigned to each study. Stata SE software (version 16.1; StataCorp, College Station, TX, USA) was used for all calculations.

## 3. Results

**Figure 1:**
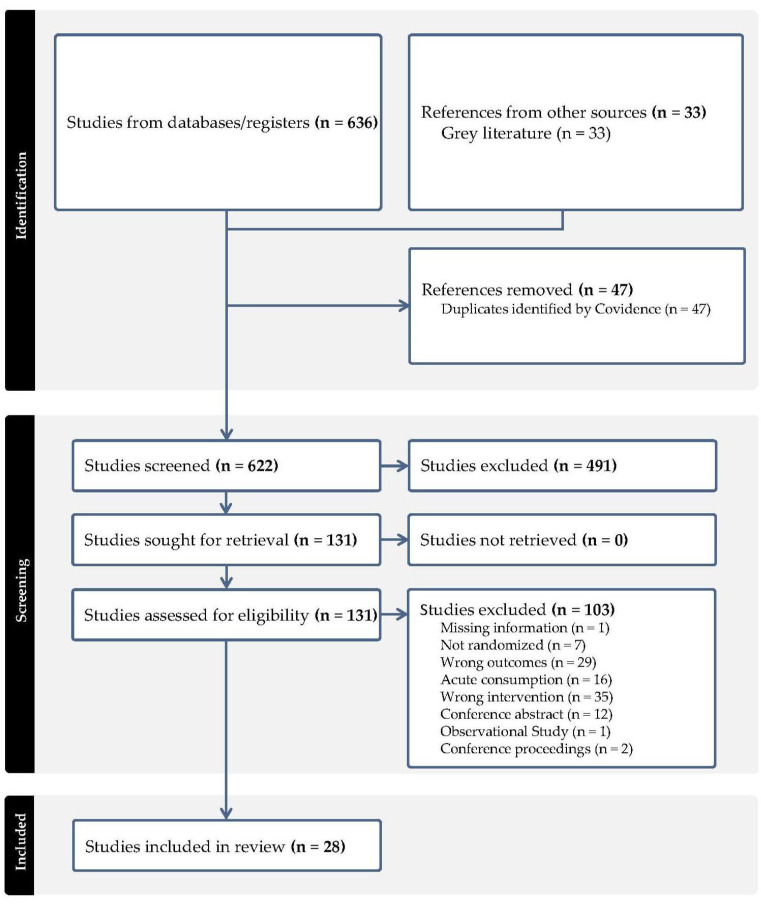
PRISMA flowchart of literature search and study selection.

The literature search identified 636 studies that were imported from screening. Out of these, 47 study duplicates were removed and 622 studies were screened, with 491 studies determined to be irrelevant. Ultimately, 131 full-text studies were assessed for eligibility, with 103 out of the 131 studies excluded primarily due to the wrong intervention unrelated to berries. A total of 28 RCTs met the eligibility criteria [29,30,31,32,33,34,35,36,37,38,39,40,41,42,43,44,45,46,47,48,49,50,51,52,53,54,55,56]. The study flow diagram is shown in Figure 1 (PRISMA).

### 3.1. Study and Subject Characteristics

A summary of the characteristics of the included RCTs is shown in Table 1. 

Individual study characteristics are shown in Table 2. The 28 studies included in the systematic review were all RCTs. The study designs used were 19 (68%) parallel RCTs [29,30,31,32,34,35,36,38,40,41,42,43,44,45,47,48,50,51,56] and 9 (32%) crossover RCTs [33,37,39,46,49,52,53,54,55]. Of the RCTs, 64% were double-blinded. Study sample sizes ranged from 10 to 138 subjects; the sample sizes in 43% of these RCTs ranged from 31 to 50 subjects. The intervention duration of the RCTs ranged from 7 days to 24 weeks. Some 71% of RCTs had an intervention duration of >4 weeks to 12 weeks.

Subjects ranged in age from 18 years to 74 years, and 57% of these subjects had a mean age of <50 years. Three out of 28 RCTs did not include females as subjects. fivesome 25 of the 28 RCTs did not report the subjects’ race. The majority (64%) of the study populations were those at risk for diseases such as cardiovascular disease and type 2 diabetes. Several studies included subjects with active disease states such as bladder cancer [48], liver disease [38] and type 2 diabetes [33]. In addition, 46% of the subjects were considered overweight, 29% were considered obese, and 25% were normal weight.

For the berry interventions, six RCTs reported using blueberries [32,35,43,47,52,54]; three RCTs each reported using acai [37,45,55], aronia (chokeberry) [51,53,56], blackcurrant [41,42,44], and cranberry [34,40,48]; and two RCTs each reported using agraz [39,46], bilberry [29,33], and mixed berries [49,50]. There was only one RCT each using maqui berry [36], pomegranate [38], strawberry [31], and whortleberry [30]. RCTs reported using differing formulations of berries. Ten (36%) RCTs used freeze-dried berry powders [29,31,35,39,40,43,46,47,52,53], ten (36%) used berry extracts [30,32,33,34,36,41,42,48,50,56], seven (25%) used berry juice [37,38,44,45,49,51,54], and one (3%) used berry pulp [55]. Regarding the differing formulations, 71% of RCTs using juice, 60% of RCTs using freeze-dried berries, and 30% of RCTs using berry extracts reported a statistically significant effect on selected biomarkers of oxidative stress, respectively. Most RCTs (79%) reported the anthocyanin content of the berry interventions, which ranged from 6.22 mg to 2250 mg (Table 2).

### 3.2. Oxidative Stress Markers Assessed

Collectively, the included RCTs reported on >10 separate biomarkers of oxidative stress. More than 68% (n = 19) of the RCTs reported results for blood antioxidants such as ferric-reducing/antioxidant power (FRAP), glutathione, oxygen radical absorbance capacity (ORAC), superoxide dismutase (SOD), total antioxidant capacity (TAC), and thiols [29,32,33,34,35,37,38,39,41,42,43,46,47,48,50,52,53,55,56]. Other commonly reported biomarkers were isoprostanes (n = 9) [34,36,39,40,44,45,53,54,56], malondialdehyde (n = 7) [30,31,32,38,41,49,55], and oxidized LDL (n = 7) [29,34,36,49,50,54,56]. Several RCTs reported on biomarkers for DNA damage such as % DNA in tail (n = 3) [33,52,55] and urine 8-oxoguanine (8-OHdG) (n = 3) [33,34,39]. Few RCTs reported using lipid peroxidation biomarkers such as thiobarbituric acid-reactive substances (TBARS) (n = 2) [39,51], protein oxidation using myeloperoxidase (n = 1) [46], and reactive oxygen species (n = 1) [41].

### 3.3. Risk of Bias

The Cochrane Risk-of-Bias assessment graded the strength of the body of evidence, which included RCTs with a parallel design (n = 19) or crossover design (n = 9). Of the 28 RCTs, 8 (29%) studies showed a low risk of bias, 17 (61%) showed some concerns for risk of bias, and 3 (10%) RCTs showed a high risk of bias. These results are presented in Table 3.

Of the 19 RCTs with a parallel design, only 2 (10%) warranted an overall high risk-of-bias judgement due to the blinding of participants, personnel, or outcome assessment, along with missing outcome data. Of the remaining studies with a parallel design, 10 (53%) RCTs had some concerns regarding risk of bias, due to several of the domains including sequence generation, allocation concealment, the blinding of participants, personnel, or the outcome assessment, and incomplete outcome data. Lastly, 7 (37%) parallel-design RCTs showed a low risk of bias.

One (11%) out of nine crossover-design RCTs showed a high risk of bias due to the blinding of participants and or personnel, along with the blinding of the outcome assessment. The majority of the crossover-design RCTs, i.e., 7 (78%), had some concerns regarding risk of bias due to sequence generation, allocation concealment, the blinding of participants, personnel, or the outcome assessment, and incomplete outcome data. One (11%) crossover design RCT showed a low risk of bias.

### 3.4. Synthesis of Results

Table 2 presents results from all included studies for changes in oxidative stress biomarkers related to berry consumption in RCTs. When berry interventions were compared with control groups, RCTs reported either statistically significant beneficial effects or no statistically significant differences in oxidative stress biomarkers. The results of biomarkers reported by three or more studies (antioxidants, DNA damage, isoprostanes, malondialdehyde and oxidized LDL) are reported below.

#### 3.4.1. Antioxidants

In 19 RCTs evaluating antioxidant biomarkers, 6 (32%) studies using interventions with acai juice, acai pulp, agraz freeze-dried powder, aronia (chokeberry) freeze-dried powder, cranberry extract, and pomegranate juice reported statistically significant increases in antioxidant capacity. Thirteen (68%) RCTs using interventions with agraz freeze-dried powder, aronia (chokeberry) extract, bilberry freeze-dried powder, bilberry extract, blackcurrant extract, blackcurrant juice, blueberry freeze-dried powder, blueberry extract, cranberry extract and a mixed berry extract of cranberry/strawberry reported no differences in antioxidant biomarkers.

#### 3.4.2. DNA Damage

In three RCTs evaluating % DNA in tail from comet assays, one (33%) study using blueberry freeze-dried powder reported statistically significant beneficial results. Two (67%) studies using acai pulp and bilberry extract reported no statistically significant differences.

In three RCTs reporting urinary 8-OHdG, one (33%) study using agraz freeze-dried powder reported statistically significant beneficial results. Two (67%) studies using bilberry extract and cranberry extract reported no statistically significant differences.

#### 3.4.3. Isoprostanes

Of nine RCTs evaluating isoprostanes, four (44%) studies using acai juice, blackcurrant juice, cranberry freeze-dried powder, and maqui berry extract reported statistically significant beneficial results. Five (56%) studies using agraz freeze-dried powder, aronia berry freeze-dried powder, aronia berry extract, blueberry juice, and cranberry extract reported no statistically significant differences.

#### 3.4.4. Malondialdehyde

In seven RCTs evaluating malondialdehyde, three (43%) studies using acai pulp, strawberry freeze-dried powder, and whortleberry extract reported statistically significant beneficial results. Four (57%) studies using blueberry extract, blackcurrant extract, a mixed berry (blueberries, blackcurrant, elderberry, lingonberries and strawberry) juice, and pomegranate juice reported no statistically significant differences.

#### 3.4.5. Oxidized LDL

In seven RCTs evaluating oxidized LDL, two (29%) studies using bilberry freeze-dried powder and maqui berry extract reported statistically significant beneficial results. Five (71%) studies using aronia berry extract, blueberry juice, cranberry extract, cranberry/strawberry extract, and a mixed berry (blueberries, blackcurrant, elderberry, lingonberries and strawberry) juice reported no statistically significant differences.

Collectively, of the 56 oxidative stress biomarkers reported by the 28 RCTs, 18 (32%) oxidative stress biomarkers were reported to have statistically significant beneficial results, and 38 (68%) oxidative stress biomarkers were reported to have no statistically significant differences.

## 4. Discussion

In this study, we conducted a systematic review to determine gaps in the literature and summarize the available evidence on the effect of ≥7 days of berry consumption on biomarkers of oxidative stress in adults over the last 10 years. The 28 RCTs identified in this systematic review reported >10 different biomarkers of oxidative stress. Biomarkers reported by three or more RCTs, including antioxidants, DNA damage, isoprostanes, malondialdehyde and oxidized LDL, were systematically reviewed.

The reviewed literature revealed several gaps. For example, only 1 RCT out of the 28 RCTs used fresh fruit in the form of berry pulp [55]; most RCTs used freeze-dried berries [29,31,35,39,40,43,46,47,52,53], berry extract [30,32,33,34,36,41,42,48,50,56], or berry juice [37,38,44,45,49,51,54]. Regarding formulations, 71% of the RCTs using juice [37,38,44,45,51], 60% of the RCTs using freeze-dried berries [29,31,39,40,52,53], and 30% of the RCTs using berry extracts [30,36,48] reported a statistically significant effect on biomarkers of oxidative stress, respectively. The majority of the RCTs using freeze-dried berries advised study subjects to reconstitute with water and to consume as a beverage. Clarification is needed as to whether berries consumed as a reconstituted freeze-dried berry beverage versus 100% juice differentially affect biomarkers of oxidative stress. The freeze-dried berry beverages included fiber, which may affect several physiological systems. The latest sequencing techniques allow for the identification of microbiota present in the intestinal tract, which leads to greater awareness of the role of fiber through its effects on the microbiota. In addition, fiber’s metabolites are thought to play a major role in the health benefits derived from fiber intake [57]. Freeze-dried berries and berry juice may be more promising interventions versus berry extracts when evaluating biomarkers of oxidative stress. Research results are conflicting, as a recent meta-analysis of RCTs showed that both dietary polyphenols from whole foods and polyphenol extracts may be effective in lowering cardiometabolic risk factors such as blood pressure, flow-mediated dilation, and lipid concentrations, though the authors suggest that results must be interpreted with caution due to high heterogeneity and risk of bias among studies [10]. The most common berry used in RCTs was the blueberry, with six studies [32,35,43,47,52,54], then açaí [37,45,55], aronia (chokeberry) [51,53,56], blackcurrant [41,42,44], and cranberry [34,40,48] in three RCTs each. Only one RCT included strawberries alone [31], though two studies included strawberries in mixed berry studies [49,50]. In recent years, there has been much interest in *E. oleracea* Mart., also known as açaí, which are native to the Amazonia and Atlantic Forest Regions and are fruits of palm trees. An integrative review of human clinical trials has suggested that açaí may contribute to improved antioxidant capacity, metabolic stress, and inflammation [58], a conclusion similar to that of the three RCTs of açaí evaluated in the current systematic review. Additionally, the intervention duration of the RCTs ranged from 7 days to 24 weeks. Some 71% of RCTs had an intervention duration of >4 weeks to 12 weeks, with only one RCT including an intervention that lasted >3 months [35].

Interestingly, almost 60% of RCTs were conducted in adults aged <50 years, even though increased oxidative stress, implicated in Alzheimer’s disease, cardiovascular diseases, and various cancers, occurs more frequently in adults aged 65 years and above. The majority (64%) of the subjects in the RCTs were those at risk for diseases such as cardiovascular disease and type 2 diabetes; 75% were either overweight or obese. Type 2 diabetes and obesity are known to alter absorption and metabolism, which may affect the bioavailability and effectiveness of polyphenols. The beneficial health effects of anthocyanins are dependent upon their bioavailability. Less than 2% of anthocyanins may be found in the blood or urine after dietary intake, which suggests low bioavailability. Anthocyanins go through several biotransformations in the small and large intestines; only a small percentage of the anthocyanins remain nonmetabolized. However, some research suggests that anthocyanin metabolites may contribute to beneficial health effects [59].

Many RCTs were conducted in North America and Europe, with few conducted in Asia, the Middle East, Oceana, and South America. No RCTs were conducted in Africa. Further, only four RCTs used a sample size calculation for biomarkers of oxidative stress [34,37,39,55].

Generally, the impact of berry consumption on oxidative stress biomarkers reported in the RCTs included here was either beneficial or null. Of the approximately 56 oxidative stress biomarkers reported by the 28 studies, 32% of the biomarkers were reported to have statistically significant beneficial results, and 68% of the biomarkers were reported as having no statistically significant differences. One out of six blueberry RCTs showed a statistically significant beneficial effect on a biomarker of oxidative stress, H_2_O_2_-induced DNA damage [52]. Three açaí RCTs reported beneficial effects on antioxidants (catalase, total antioxidant capacity and glutathione), isoprostanes and malondialdehyde [37,45,55]; two out of three aronia (chokeberry) RCTs reported favorable effects on TBARS [51] and glutathione [53]; one out of the three blackcurrant RCTs reported a significant decrease in isoprostanes [44]; and two out of three cranberry RCTs reported beneficial changes in isoprostanes [40] and antioxidants (SOD and TAC) [48].

The large number of biomarkers of oxidative stress reported in the RCTs reviewed render the identification of clear antioxidant effects of berries difficult. Further complicating this endeavor are the different methodologies used to measure biomarkers (e.g., mass spectroscopy vs. enzyme-linked immunoassay kits) and the fact that individual RCTs include multiple biomarkers. RCTs in this and another recent review predominantly reported on blood antioxidants, isoprostanes, malondialdehyde, and oxidized LDL [6]. Isoprostane analysis via liquid chromatography tandem mass spectrometry is an established and accurate methodology [60,61,62,63]. Analysis of antioxidant capacity via electrochemical/chemical methods and oxidized LDL via isolation and fractionation are also well described [64,65,66]. Moving forward toward a more standardized reporting of oxidative stress biomarkers and perhaps consistency across RCTs would require investigators to select a small number of biomarkers linked with human health outcomes that may be measured using the most accurate methods. For example, F2-isoprostane levels have been shown to be associated with metabolic syndrome, and are optimally measured using liquid chromatography tandem mass spectrometry assays [61,63].

A factor contributing to null findings across RCTs may be inadequate power, as only 4 (14%) out of 28 studies determined a sample size calculation for biomarkers of oxidative stress. RCTs should have enough power to show the potential effect of the intervention. A recent statistical commentary provided guidance for efficient sample size determination for RCTs, focusing on parallel and crossover designs [67]. For the current systematic review, two out of four RCTs that reported a sample size calculation found statistically significant results. De Liz et al. reported that consuming 200 mL acai juice, containing 100 mg anthocyanins, for 12 weeks increased catalase, TAC, and glutathione in healthy subjects [37]. Espinosa-Moncada et al. found that after 4 weeks of consuming 200 g of agraz (Andean berry), containing 76 mg anthocyanins, TAC was significantly increased and 8-OHdG was significantly decreased in males with metabolic syndrome [39]. However, Chew et al. found no statistically significant results for 8-OHdG in obese subjects consuming a 450 mL cranberry extract beverage, containing 6.22 mg anthocyanins, daily for 8 weeks [34]. Interestingly, Terrazas et al. reported a sample size calculation for DNA damage with no statistically significant effects in healthy male cyclists consuming 400 g açai pulp, containing 71 mg anthocyanins, for 15 days [55]. However, the same study showed that açai pulp decreased lipid peroxidation (malondialdehyde) and increased antioxidant capacity. This increased antioxidant capacity, which provides the first line of antioxidant defense mechanisms, may also mediate effects on lipid peroxidation [3]. 

Another factor contributing to the lack of consistent findings across RCTs may be related to differences in intestinal microbiota profiles. Microbial transformation of dietary polyphenols, including anthocyanins, into secondary metabolites is important to the bioavailability and bioactivity of polyphenols [68,69,70,71]. Evidence also shows that dietary polyphenols modify the intestinal microbiota population. Some studies have demonstrated a significant mediating effect of microbiota on outcomes such as blood pressure, inflammatory status, and intestinal integrity [72,73,74,75]. Despite the known association of microbiota characteristics with the health outcomes of polyphenol-rich diets, none of the studies we reviewed included intestinal microbiota as a measured outcome variable. In a previous systematic review, we presented the limited number of RCTs that included microbiota as a factor in the relationship between polyphenol intake and blood pressure [76]. We suggest that results of RCTs testing the effect of berries on oxidative stress biomarkers might be more consistent if the microbiota profiles of participants were assessed and included in the analysis. Another limitation is the lack of stratification by sex in the articles reviewed. Biomarkers of oxidative stress have been shown to be higher in females than males [77].

The strengths and limitations of this study design warrant consideration. To our knowledge, this is the first systematic review of RCTs in humans evaluating the effects of berry consumption on biomarkers of oxidative stress in the last 10 years. RCTs are considered the optimal study design to determine causality [78]. We carefully followed the rigorous methodology of the Cochrane collaboration for systematic reviews for interventions [79]. The search strategy applied was thorough, and involved computerized and manual searches. In addition, Covidence, Veritas Health Innovation, Melbourne, Australia, available at www.covidence.org (an online systematic review software for deduplication, screening, data extraction, and quality assessment) was utilized, which assists with efficiency in conducting systematic reviews and may ultimately decrease researcher errors. The primary limitation of this study is the heterogeneity across RCTs, such as differing berry interventions and preparations, varying subject characteristics, and differing methodologies that measure oxidative stress biomarkers, and a lack of sample size determination for oxidative stress biomarkers; therefore, caution should be used when interpreting results and conclusions.

## 5. Conclusions

Overall, evidence reviewed in the study suggests that 32% of oxidative stress biomarkers reported by the 28 RCTs showed statistically significant beneficial results, and 68% oxidative stress biomarkers showed no statistically significant differences. More well-designed and longer-term berry RCTs are needed to evaluate biomarkers of oxidative stress, especially in females and older adults, utilizing a sample size calculation for determining study power.

## Figures and Tables

**Table 1:** Study characteristics and most common biomarkers of oxidative stress reported by the 28 randomized controlled trials ^1^.

Study Characteristics	Total	Antioxidants	Isoprostanes	MDA	OxLDL
n	28	19	9	7	7
Study design, n (% of studies)					
Parallel	19 (68%)	12 (63%)	6 (67%)	5 (71%)	5 (71%)
Crossover	9 (32%)	7 (37)	3 (33%)	2 (29%)	2 (29%)
Blinding					
Double-blind	18 (64%)	12 (63%)	8 (89%)	3 (43%)	4 (57 %)
Single-blind	4 (14%)	2 (11%)	1 (11%)	2 (29%)	1 (14%)
Not blinded	3 (11%)	2 (11%)	0 (0%)	2 (29%)	2 (29%)
Unclear	3 (11%)	3 (15%)	0 (0%)	0 (0%)	0 (0%)
Region, n (% of studies)					
Asia	1 (4%)	1 (5%)	0 (0%)	0 (0%)	0 (0%)
New Zealand	1 (4%)	1 (5%)	0 (0%)	1 (14%)	0 (0%)
Europe	9 (32%)	5 (26%)	3 (33%)	2 (29%)	3 (43%)
North America	10 (36%)	6 (32%)	5 (56%)	1 (14%)	4 (57%)
South America	4 (14%)	4 (21%)	1 (11%)	1 (14%)	0 (0%)
Middle East	3 (11%)	2 (11%)	0 (0%)	2 (29%)	0 (0%)
Sample size, n (% of studies)					
10–20	4 (14%)	3 (15%)	1 (11%)	1 (14%)	1 (14%)
21–30	3 (11%)	3 (15%)	0 (0%)	1 (14%)	0 (0%)
31–50	12 (43%)	7 (38%)	4 (44%)	2 (29%)	3 (43%)
51–100	7 (25%)	4 (21%)	3 (33%)	3 (43%)	3 (43%)
>100	2 (7%)	2 (11%)	1 (11%)	0 (0%)	0 (0%)
Intervention duration, n (% of studies)					
≥1 week to 4 weeks	7 (25%)	3 (15%)	1 (11%)	1 (14%)	1 (14%)
>1 month to 3 months	20 (71%)	12 (64%)	7 (78%)	6 (86%)	5 (71%)
>3 months	1 (4%)	4 (21%)	1 (11%)	0 (0%)	1 (14%)
Mean age, n (% of studies)					
<50 years	16 (57%)	10 (53%)	6 (67%)	5 (71%)	2 (29%)
≥50 years	12 (43%)	9 (47%)	3 (33%)	2 (29%)	5 (71%)
Baseline health, n (% of studies)					
Healthy	10 (36%)	7 (38%)	3 (33%)	4 (57%)	3 (43%)
At-risk	18 (64%)	12 (62%)	6 (67%)	3 (43%)	4 (57%)
Weight status, n (% of studies)					
Normal weight: BMI 18.5 kg/m² and 25 kg/m²	7 (25%)	5 (26%)	0 (0%)	3 (43%)	1 (14%)
Overweight: BMI 25 kg/m² and 29.9 kg/m²	13 (46%)	10 (53%)	5 (56%)	3 (43%)	3 (43%)
Obese: BMI 30 kg/m² or higher	8 (29%)	4 (21%)	4 (44%)	1 (14%)	3 (43%)
Intervention, n (% of studies)					
Acai	3 (11%)	2 (11%)	1 (11%)	1 (14%)	0 (0%)
Agraz	2 (7%)	2 (11%)	1 (11%)	0 (0%)	0 (0%)
Aronia (Chokeberry)	3 (11%)	2 (11%)	2 (22%)	0 (0%)	1 (14%)
Bilberry	2 (7%)	2 (11%)	0 (0%)	0 (0%)	1 (14%)
Blackcurrant	3 (11%)	2 (11%)	1 (11%)	1 (14%)	0 (0%)
Blueberry	6 (21%)	5 (26%)	1 (11%)	1 (14%)	1 (14%)
Cranberry	3 (11%)	2 (11%)	2 (22%)	0 (0%)	1 (14%)
Maqui berry	1 (4%)	0 (0%)	1 (11%)	0 (0%)	1 (14%)
Pomegranate	1 (4%)	1 (5%)	0 (0%)	1 (14%)	0 (0%)
Strawberry	1 (4%)	0 (0%)	0 (0%)	1 (14%)	0 (0%)
Whortleberry	1 (4%)	0 (0%)	0 (0%)	1 (14%)	0 (0%)
Mixed berries	2 (7%)	1 (5%)	0 (0%)	1 (14%)	2 (29%)

^1^ Values of 0 (0%) show potential gaps in the literature, where no randomized controlled trial met the provided study characteristic. Abbreviations: MDA, malondialdehyde; Ox-LDL, oxidized low-density lipoprotein.

**Table 2:** Study and subject characteristics with results for biomarkers of oxidative stress reported by the 28 randomized controlled trials.

Author, Year, Country	Study Funding Sources	Study Design	Duration of Study Intervention	Total n Enrolled; Total n Female; Dropout Rate (%)	Mean (SD) Age, Years	Health Status	Mean (SD) BMI (kg/m); Weight Status	Berry Intervention; Formulation; Amount	Control Group	Oxidative Stress Bio-Markers	Key Findings for Berries vs. Control Group	Sample Size Calculation
Arevström et al., 2019, Sweden [29]	Örebro University Hospital Research Foundation	Parallel	8 weeks	55; NR; 9%	68 (62–74) median (IQR)	Post acute myocardial infarction (AMI); at risk	~27.7 (5.5); overweight	Bilberry (*Vaccinium myrtillus*); freeze-dried powder; 40 g, equivalent to 480 g fresh bilberries, 2250 mg anthocyanins	Post AMI standard medical nutrition therapy	Serum ox-LDL Serum antioxidant capacity	Compared to the control group, ox-LDL was significantly decreased (~20%) in the bilberry group (*p* = 0.017). Differences in antioxidant activity between groups were not statistically significant.	High sensitivity C-reactive protein
Asgary et al., 2016, Iran [30]	None declared	Parallel	4 weeks	54; NR; 7%	47 (16)	Hyperlipidemia; at risk	25.3 (1.9); overweight	Caucasian whortleberry (*Vaccinium arctostaphylos* L.); extract; 2 capsules, 500 mg dried granules, 90 mg anthocyanins	2 placebo capsules	Serum MDA	Compared to placebo, Vaccinium arctistaphylos fruit extract significantly decreased MDA (*p* = 0.013).	NR
Basu et al., 2014, USA [31]	California Strawberry Commission; National Institutes of Health	Parallel	12 weeks	66; NR; 9%	49 (10)	Abdominal adiposity and dyslipidemia (elevated serum lipids); at risk	~36.0 (4.8); obese	Strawberries (cultivars Camarosa (37%), Ventana (13%), Diamante (13%), and 2 proprietary varieties (37%); freeze-dried powder; 25 g, 78 mg anthocyanins and 50 g, 155 mg anthocyanins	Isocaloric placebo powder (sugar and matched for fiber) without poly-phenols	Serum MDA and HNE	After 12 weeks, MDA and HNE were significantly decreased in the 25 g group vs. the 25 g control group (*p* < 0.01) and in the 50 g group vs. the 50 g control group (*p* < 0.001).	Total cholesterol and LDL cholesterol
Bowtell et al., 2017, United Kingdom [32]	Alzheimer Charity BRACE; Cherry Active Ltd	Parallel	12 weeks	26; 13; NR	~68 (1)	Healthy	~26.5 (1.4) overweight	Blueberry concentrate; extract; 30 mL, 387 mg anthocyanins	30 mL isocaloric placebo: a synthetic blackcurrant and apple cordial (Robinsons cordial, Britvic Ltd., Hemel Hempstead, UK) with sugar added to match blueberry energy content	Serum Glutathione Serum MDA Serum HNE protein carbonylation	Compared to baseline, serum glutathione concentration was significantly decreased in both the placebo group and blueberry group (main time effect *p* < 0.001). The blueberry group experienced a smaller decrease (placebo: −11.7 ± 2.8%; blueberry: −6.5 ± 2.4%; *p* = 0.09). No statistically significant differences in MDA or HNE protein carbonylation.	NR
Chan et al., 2021, China [33]	Caritas Institute of Higher Education	Cross-over	4 weeks	20; 11; 0%	~56 (10)	Type 2 diabetes; at risk	~27.2 (4.2); overweight	European Bilberry (*Vaccinium vitis-idaea* L.); extract; 4 capsules, 1.4 g anthocyanins	4 placebo capsules	Erythrocyte SOD Erythrocyte GPx Plasma FRAP Urinary 8-oxoguanine H_2_O_2_-induced DNA damage (Comet assay)	No statistically significant differences.	Hemo-globin A1C, plasma ascorbic acid, uric acid, total cholesterol and LDL cholesterol
Chew et al., 2019, USA [34]	Ocean Spray Cranberries Inc.; USDA	Parallel	8 weeks	79; NR; 1%	43 (1)	Healthy, non-smokers	30.8 (0.4); obese	Cranberry (*Vaccinium macrocarpon*); extract beverage; 450 mL, 6.22 mg anthocyanins	450 mL isocaloric placebo beverage without poly-phenols	Erythrocyte GSH Erythrocyte GSSG Erythrocyte GPx Erythrocyte SOD Urinary F2-isoprostanes Urinary 8-OHdG Plasma ox-LDL	No statistically significant differences.	8-OHdG
Curtis et al., 2019, United Kingdom [35]	US Highbush Blueberry Council	Parallel	6 months	138; NR; 17%	63 (7)	Metabolic syndrome; at risk	31.2 (3.0); obese	Blueberry (*Vaccinium corymbosum* L.); freeze-dried powder; 26 g equivalent to 1 cup fresh blueberries, 364 mg anthocyanins and 13 g equivalent to 1/2 cup fresh blueberries, 182 mg anthocyanins	Isocaloric placebo powder without poly-phenols	Total free thiols in plasma	No statistically significant differences.	Homeo-stasis model assessment of insulin resistance
Davinelli et al., 2015, Italy [36]	Enervit S.p.A; Maqui New Life Inc.	Parallel	4 weeks	42; 13; 0%	NR; 45–65 years inclusion criteria	Smokers; at risk	~28.7 (4.4); overweight	Maqui berry (*Aristotelia chilensis*), Delphinol™; extract; 3 capsules, 162 mg anthocyanins	3 placebo capsules	Plasma ox-LDL Urinary F2-isoprostanes	Compared to baseline, there were significant decreases in ox-LDL and F2-isoprostanes in the treatment group after 4 weeks of Delphinol maqui berry extract consumption (*p* < 0.05).	NR
deLiz et al., 2020, Brazil [37]	National Council for Scientific and Technological Development; Ministry of Education, Brazil	Cross-over	12 weeks	38; NR; 21%	~64 (13)	Healthy	22.2 (2.5); normal weight	Açaí (*E. oleracea*); juice; 200 mL, 100 mg anthocyanins	Juçara (*E. edulis*); juice; 200 mL, 627 mg anthocyanins	Erythrocyte GPx Erythrocyte CAT Erythrocyte SOD Serum TOS Serum TAC OSI (TOS:TAC)	Açaí juice intake caused significant increases in TAC activity, CAT activity, GPx activity and OSI compared to baseline (*p* < 0.05). Juçara juice intake caused a significant increase in CAT activity compared to baseline (*p* < 0.05) with no statistically significant effects on TAC activity, GPx activity and OSI.	CAT, TAC, total cholesterol, LDL-cholesterol and tri-glycerides
Ekhlasi et al., 2015, Iran [38]	Iran University of Medical Sciences	Parallel	12 weeks	70; 21; 7%	39 (8)	Non-alcoholic fatty liver disease, metabolic syndrome; at risk	29.7 (2.7); overweight	Pomegranate (*Punica granatum*); juice, 250 mL	Orange juice, 250 mL	Plasma TAC Plasma MDA	Compared to baseline, TAC increased in both the pomegranate juice group (*p* < 0.01) and orange juice group (*p* < 0.05). The mean change in TAC was significantly greater in the pomegranate group (*p* = 0.03). No statistically significant differences for MDA.	NR
Espinosa-Moncada et al., 2018, Colombia [39]	Departamento Administrativo de Ciencia, Tecnologíae Innovación Colciencias; University of Antioquia UdeA, Medellin-Colombia	Cross-over	4 weeks	40; 40; 0%	47 (9)	Metabolic syndrome; at risk	~29.8 (4.1); overweight	Agraz or Andean berry (*Vaccinium meridionale* Swartz); freeze-dried powder; equivalent to 200 g fresh fruit, 76 mg anthocyanins	Isocaloric placebo without poly-phenols	TBARS Serum antioxidant capacity Urinary F2-isoprostanes Urinary 8-OHdG	After 4 weeks, serum antioxidant capacity was significantly increased (*p* = 0.028) and 8-OHdG was significantly lower (*p* = 0.041) in the treatment group compared to the placebo group. No statistically significant differences for TBARS and F2-isoprostanes.	Blood pressure, plasma tri-glyceride, LDL cholesterol, oxidative stress, and inflammatory biomarkers
Hsia et al., 2020, USA [40]	Ocean Spray Cranberries Inc; National Institute of General Medical Sciences of the National Institutes of Health; Nutrition Obesity Research Center	Parallel	8 weeks	37; NR; 5%	~48 (14)	At-risk for CVD, metabolic syndrome, elevated fasting glucose or impaired glucose tolerance; at risk	~36.9 (2.7); obesity	Cranberry (*Vaccinium macrocarpon*); freeze-dried powder; beverage 450 mL, 6.5 mg anthocyanins	450 mL isocaloric placebo beverage without poly-phenols	8-isoprostanes ox-LDL MDA	Changes in 8-isoprostane were significantly different between groups: –2.18 (cranberry) vs. +20.81 (placebo) (*p* = 0.02). No statistically significant differences for ox-LDL and MDA.	Insulin sensitivity
Hurst et al., 2020, New Zealand [41]	New Zealand Ministry for Business Innovation and Employment	Parallel	5 weeks	36; NR; 6%	38 (11)	Healthy	~24.8 (2.8); normal weight	New Zealand blackcurrant (*Ribes nigrum*); extract; 2 capsules, 240 mg anthocyanins	2 placebo capsules	Plasma MDA Plasma reactive oxygen species-generating capability Plasma superoxide-generating capability Plasma FRAP	No statistically significant differences.	NR
Hutchison et al., 2016, USA [42]	None reported	Parallel	7 days	24; 18; 33%	~20 (1)	Healthy	~23.2 (2.2); normal weight	Blackcurrant (*Ribes nigrum*); extract beverage; 480 mL, 369 mg anthocyanins	Isocaloric placebo beverage without polyphenols	Plasma ORAC	No statistically significant differences.	Pain
Johnson et al., 2015, USA [43]	US Highbush Blueberry Council/US Department of Agriculture	Parallel	8 weeks	48; 48; 17%	~59 (5)	Pre- and stage 1-hyper-tension; at risk	~31.4 (6.4); obese	Blueberry (50/50 blend of tifblue (*Vaccinium virgatum*) and rubel (*Vaccinium corymbosum*); freeze-dried powder; 22 g; 470 mg anthocyanins	Isocaloric placebo powder without poly-phenols	Serum SOD	No statistically significant differences.	Blood pressure
Khan et al., 2014, Scotland [44]	GlaxoSmithKline	Parallel	6 weeks	66; 22; 3%	~53 (9)	Healthy	~28.8 (6.3); overweight	Blackcurrant (*Ribes nigrum*); low (6.4% juice) drink and high (20% juice) drink; 250 mL, low drink, 10 mg anthocyanins and high drink, 36 mg anthocyanins	250 mL placebo flavored water beverage	Plasma F2-isoprostanes	After 6 weeks, F2-isoprostanes were significantly decreased in the high blackcurrant juice group compared to the low blackcurrant juice group (*p* = 0.002) and the placebo group (*p* = 0.003).	Flow-mediated dilation
Kim et al., 2018, USA [45]	None stated	Parallel	12 weeks	43; NR; 14%	~44 (13)	Metabolic syndrome; at risk	~33.5 (6.2); obese	Açaí (*E. oleracea*); juice; 650 mL, 307 mg anthocyanins	650 mL placebo beverage	Urinary 8-isoprostanes	After 12 weeks of açaí beverage consumption, 8-isoprostanes were significantly decreased by 31.2% compared to the placebo group (*p* = 0.0099).	C-reactive protein
Marín-Echeverri et al., 2018, Colombia [46]	Departamento Administrativo de Ciencia, Tecnologíae Innovación; University of Antioquia UdeA, Medellin-Colombia	Cross-over	12 weeks	40; 40; 0%	47 (9)	Metabolic syndrome; at risk	>25; overweight	Agraz or Andean berry (*Vaccinium meridionale* Swartz); freeze-dried powder; equivalent to 200 g fresh agraz	Isocaloric placebo powder without poly-phenols	Serum MPO Serum advanced oxidation protein products	No statistically significant differences.	NR
McAnulty et al., 2014, USA [47]	Appala-chian State University; US Highbush Blueberry Council	Parallel	6 weeks	25; NR; NR	40 (14)	Healthy, pre-hyper-tensive; at risk	27.8 (5.5); overweight	Blueberry (50/50 blend of tifblue (*Vaccinium virgatum*) and rubel (*Vaccinium corymbosum*)); freeze-dried powder; 38 g equivalent to 250 g fresh fruit	Isocaloric placebo powder without poly-phenols	Plasma ORAC Plasma FRAP	No statistically significant differences.	NR
Mohammed et al., 2016, Iraq [48]	None	Parallel	6 weeks	45; 12; 11%	Range: 60–70 years; mean and SD NR	Bladder cancer; at risk	>25; overweight	American cranberry (*Vaccinium macrocarpon*); extract; 2 capsules containing 36 mg proanthocyanidins	2 placebo capsules	Serum SOD1 Serum TAC	Following treatment, SOD1 and TAC were significantly increased in the cranberry group than in the placebo group (*p* < 0.05).	NR
Nilsson et al., 2017, Sweden [49]	Lund University	Cross-over	5 weeks	46; NR; 11%	63 (1)	Healthy	24.4 (0.4); normal weight	Mixed berries; juice; 150 g blueberries, 50 g blackcurrant, 50 g elderberry, 50 g lingonberries, 50 g strawberry, and 100 g tomatoes, 600 mL, 795 mg polyphenols	600 mL isocaloric beverage without poly-phenols	Plasma MDA Serum ox-LDL	No statistically significant differences.	Verbal working memory test
Paquette et al., 2017, Canada [50]	Consortium de recherche et innovations en bio-procédés industriels au Québec, Atrium Innovations Inc. and Nutra Canada	Parallel	6 weeks	50; NR; 8%	~59 (7)	Insulin resistant, impaired fasting plasma glucose; at risk	~31.1 (7.1); obese	Strawberry (*Fragaria × ananassa* Duch) and cranberry (*Vaccinium macrocarpon* L.); extract; 120 mL, 333 mg poly-phenols	120 mL placebo flavored water beverage	Ox-LDL Plasma FRAP	No statistically significant differences.	Insulin sensitivity
Petrovic et al., 2016, Serbia [51]	Ministry of Education, Science and Technological Development of the Republic of Serbia. Nutrika d.o.o. Belgrade, Serbia	Parallel	4 weeks	32; 17; NR	~18 (1)	Healthy; non-smokers; handball players	~22.8 (4.0); normal weight	Aronia (*Aronia melanocarpa*), chokeberry; juice; 100 mL, 587 mg polyphenols	100 mL isocaloric beverage without poly-phenols	Plasma TBARS	After chokeberry juice consumption, TBARS levels were significantly decreased in male handball players (*p* < 0.05), but not in female handball players.	NR
Riso et al., 2013, Italy [52]	Cariplo Foundation; Wild Blueberry Association of North America; Future Ceuticals	Cross-over	6 weeks	20; 0; 10%	48 (10)	At risk of CVD; at risk	24.8 (2.6); normal weight	Blueberry (*Vaccinium angustifolium*); freeze-dried powder; 25 g equivalent to 1 cup fresh fruit, 375 mg anthocyanins	Isocaloric placebo powder without poly-phenols	Erythrocyte SOD activity Erythrocyte GSH-Px activity Lymphocyte GST activity H_2_O_2_-induced DNA damage (Comet assay)	Levels of H_2_O_2_-induced DNA damage were significantly decreased in the wild blueberry drink group (*p* ≤ 0.01), but not in the placebo group (*p* = 0.84). No significant differences for SOD, GSH-Px, and GST.	Reactive hyperemia index
Sangild et al., 2023, Denmark [53]	Ministry of Food, Agriculture and Fisheries of Denmark	Cross-over	12 weeks	109; 0; 13%	~50 (10)	Healthy, hyper-lipidemia, mildly hyper-cholesterolemic, smokers and non-smokers; at risk	~27.3 (3.3); overweight	Aronia, chokeberry (*A. arbutifolia* var. Virinia (9%), *A. prunifolia* var. Vikilia (9%), and *A. melanocarpa* var. Swecia (3%) and the cultivated *Aronia hybrid × Sorbaronia mitschurinii* var Roar (79%)); freeze-dried powder; 9 capsules per day equivalent to 150 mg total anthocyanins	9 placebo capsules	Plasma Isoprostanes Plasma Glutathione Plasma CAT Plasma SOD	Compared to placebo, Aronia intake significantly increased glutathione levels (*p* ≤ 0.05). No significant differences for SOD, CAT, and isoprostanes.	HDL cholesterol
Stote et al., 2017, Canada [54]	Wild Blueberry Association of North American	Cross-over	2 one-week treatment periods separated by an 8-day washout period	20; 20; 5%	53 (6.3)	Prediabetic, pre and stage 1 hyper-tension, hyper-lipidemia, insulin resistance, insulin sensitive; at risk	31.4 (2.9); obese	Blueberry (*Vaccinium angustifolium*); juice; 240 mL, 314 mg anthocyanins	240 mL isocaloric beverage without poly-phenols	Plasma total 8-isoprostanes Plasma ox-LDL	No statistically significant differences.	Systolic blood pressure
Terrazas et al., 2020, Brazil [55]	Petruz Fruity Group; Coordenação de Aperfeiço-amento de Pessoal de Nível Superior, Brasil	Cross-over	Two 15-day trials (1 and 2), within a 30-day wash-out between trials	10; 0; 0%	34 (5)	Healthy; male cyclists	23.9 (1.4); normal weight	Açai (*Euterpe oleracea Martius*); pulp; 400 g, 71 mg anthocyanins	400 g placebo pulp without poly-phenols	Serum MDA Serum Antioxidant capacity (TEAC) DNA damage in peripheral blood (Comet assay)	Açai pulp intervention decreased serum lipid peroxidation (MDA) by 8.53% (*p* = 0.005) and increased serum antioxidant capacity (*p* = 0.005). No significant effect on DNA damage.	DNA damage
Xie et al., 2017, USA [56]	Connecticut Department of Public Health	Parallel	12 weeks	56; 30; 12.5%	~35 (19)	Healthy; former smokers	~26.3 (7.5); overweight	Aronia chokeberry (*Aronia melanocarpa*); extract; 500 mg, 2 capsules, 45 mg anthocyanins	2 placebo capsules	Plasma ox-LDL Plasma TAC Plasma CAT Plasma GPx Plasma SOD Urinary 8-isoprostanes	No statistically significant differences.	LDL cholesterol

Abbreviations: CAT, catalase; FRAP, ferric-reducing/antioxidant power; GPx, glutathione peroxidase; GSH, reduced glutathione; GSSG, oxidized glutathione; HNE, hydroxynonenal; MDA, malondialdehyde; ORAC, oxygen radical absorbance capacity; OSI, oxidative stress index; ox-LDL, oxidized low-density lipoprotein; SOD, superoxide dismutase; TAC, total antioxidant capacity; TBARS, thiobarbituric acid reactive substances; TOS, total oxidant status; 8-OHdG, 8-hydroxydeoxyguanosine. If total mean ± SD was not reported for subjects, the means are reported as calculated weighted averages as indicated by ~ symbol. Weight status categories are based on body mass index (BMI): normal-weight BMI = 18.5–24.9, overweight BMI = 25.0–29.9, obese BMI = ≥30.0.

**Table 3:** Cochrane assessment of risk of bias.

Author, Year, Country	Sequence Generation ^1^	Allocation Concealment ^2^	Blinding of Participants and Personnel ^3^	Blinding of Outcome Assessment ^4^	Incomplete Outcome Data ^5^	Overall Risk-of-Bias Judgement ^6^
Arevström et al., 2019, Sweden [29]	Low risk	Unclear	High risk	Low risk	High risk	High risk of bias
Asgary et al., 2016, Iran [30]	Low risk	Unsure	Low risk	Unsure	Low risk	Some concerns
Basu et al., 2014, USA [31]	Unsure	Unsure	Low risk	Low risk	Low risk	Some concerns
Bowtell et al., 2017, United Kingdom [32]	Low risk	Unsure	Low risk	Low risk	Unsure	Some concerns
Chan et al., 2021, China [33]	Low risk	Unsure	Low risk	Unsure	Low risk	Some concerns
Chew et al., 2019, USA [34]	Low risk	Low risk	Low risk	Low risk	Low risk	Low risk of bias
Curtis et al., 2019, United Kingdom [35]	Low risk	Low risk	Low risk	Low risk	Low risk	Low risk of bias
Davinelli et al., 2015, Italy [36]	Unsure	Unsure	Low risk	Low risk	Low risk	Some concerns
deLiz et al., 2020, Brazil [37]	Low risk	Low risk	Low risk	High risk	Low risk	Some concerns
Ekhlasi et al., 2015, Iran [38]	Unsure	Unsure	High risk	High risk	Low risk	High risk of bias
Espinosa-Moncada et al., 2018, Colombia [39]	Low risk	Unsure	Low risk	Unsure	Unsure	Some concerns
Hsia et al., 2020, USA [40]	Low risk	Low risk	Low risk	Low risk	Low risk	Low risk of bias
Hurst et al., 2020, New Zealand [41]	Low risk	Low risk	Low risk	Unsure	High risk	Some concerns
Hutchison et al., 2016, USA [42]	Low risk	Low risk	Low risk	Low risk	Low risk	Low risk of bias
Johnson et al., 2015, USA [43]	Low risk	Low risk	Low risk	Low risk	Low risk	Low risk of bias
Khan et al., 2014, Scotland [44]	Low risk	Low risk	Low risk	Low risk	Low risk	Low risk of bias
Kim et al., 2018, USA [45]	Unsure	Unsure	Low risk	Low risk	Low risk	Some concerns
Marín-Echeverri et al., 2018, Colombia [46]	Unsure	Unsure	Low risk	Low risk	Unsure	Some concerns
McAnulty et al., 2014, USA [47]	Low risk	Unsure	Unsure	Unsure	Unsure	Some concerns
Mohammed et al., 2016, Iraq [48]	Unsure	Unsure	Unsure	Unsure	Low risk	Some concerns
Nilsson et al., 2017, Sweden [49]	Low risk	Unsure	High risk	High risk	Low risk	High risk of bias
Paquette et al., 2017, Canada [50]	Low risk	Low risk	Low risk	Low risk	Low risk	Low risk of bias
Petrovic et al., 2016, Serbia [51]	Unsure	Unsure	Low risk	Low risk	Unsure	Some concerns
Riso et al., 2013, Italy [52]	Low risk	Unsure	Low risk	Unsure	Low risk	Some concerns
Sangild et al., 2023, Denmark [53]	Low risk	Low risk	Low risk	Low risk	Low risk	Low risk of bias
Stote et al., 2017, Canada [54]	Low risk	Low risk	Low risk	Unsure	Low risk	Some concerns
Terrazas et al., 2020, Brazil [55]	Unsure	Unsure	Unsure	Unsure	Unsure	Some concerns
Xie et al., 2017, USA [56]	Low risk	Unsure	Low risk	Unsure	Low risk	Some concerns

^1^ Describe the method used to generate the allocation sequence in sufficient detail to allow an assessment of whether it should produce comparable groups. ^2^ Describe the method used to conceal the allocation sequence in sufficient detail to determine whether intervention allocations could have been foreseen in advance of, or during, enrollment. ^3^ Describe all measures used, if any, to blind study participants and personnel from knowledge of which intervention a participant re-ceived. Provide any information relating to whether the intended blinding was effective. ^4^ Describe all measures used, if any, to blind outcome assessors from knowledge of which intervention a participant received. Provide any information relating to whether the intended blinding was ef-fective. ^5^ Describe the completeness of outcome data for each main outcome, including attrition and exclusions from the analysis. State whether attrition and exclusions were reported, the numbers in each intervention group (compared with total randomized participants), reasons for attri-tion/exclusions where reported, and any re-inclusions in analyses performed by the review au-thors. ^6^ Overall risk-of-bias judgement criteria, low risk: the study is judged to be at a low risk of bias for all five domains; some concerns: the study is judged to raise some concerns in at least one domain for the result, but not to be at high risk of bias for any domain; and high risk: the study is judged to be at high risk of bias in at least one domain for the result, or not to be at high risk of bias for any domain or the study is judged to have some concerns for multiple domains in a way that substancially lowers confidence in the result.

## Data Availability

The datasets analyzed in this study are available within the article and may be obtained from the corresponding author.

## References

[1] Bakhtina A.A., Pharaoh G.A., Campbell M.D., Keller A., Stuppard R.S., Marcinek D.J., Bruce J.E. (2023). Skeletal muscle mitochondrial interactome remodeling is linked to functional decline in aged female mice. Nat. Aging.

[2] Hajam Y.A., Rani R., Ganie S.Y., Sheikh T.A., Javaid D., Qadri S.S., Pramodh S., Alsulimani A., Alkhanani M.F., Harakeh S. (2022). Oxidative Stress in Human Pathology and Aging: Molecular Mechanisms and Perspectives. Cells.

[3] Valko M., Leibfritz D., Moncol J., Cronin M.T., Mazur M., Telser J. (2007). Free radicals and antioxidants in normal physiological functions and human disease. Int. J. Biochem. Cell Biol..

[4] Vatner S.F., Zhang J., Oydanich M., Berkman T., Naftalovich R., Vatner D.E. (2020). Healthful aging mediated by inhibition of oxidative stress. Ageing Res. Rev..

[5] Aune D. (2019). Plant Foods, Antioxidant Biomarkers, and the Risk of Cardiovascular Disease, Cancer, and Mortality: A Review of the Evidence. Adv. Nutr..

[6] Avila-Escalante M.L., Coop-Gamas F., Cervantes-Rodriguez M., Mendez-Iturbide D., Aranda-González I.I. (2020). The effect of diet on oxidative stress and metabolic diseases-Clinically controlled trials. J. Food Biochem..

[7] Satija A., Bhupathiraju S.N., Spiegelman D., Chiuve S.E., Manson J.E., Willett W., Rexrode K.M., Rimm E.B., Hu F.B. (2017). Healthful and Unhealthful Plant-Based Diets and the Risk of Coronary Heart Disease in U.S. Adults. J. Am. Coll. Cardiol..

[8] Del Rio D., Rodriguez-Mateos A., Spencer J.P., Tognolini M., Borges G., Crozier A. (2013). Dietary (poly)phenolics in human health: Structures, bioavailability, and evidence of protective effects against chronic diseases. Antioxid. Redox Signal..

[9] U.S. Department of Agriculture ARS (2019). FoodData Central. https://fdc.nal.usda.gov/.

[10] Kiyimba T., Yiga P., Bamuwamye M., Ogwok P., Van der Schueren B., Matthys C. (2023). Efficacy of Dietary Polyphenols from Whole Foods and Purified Food Polyphenol Extracts in Optimizing Cardiometabolic Health: A Meta-Analysis of Randomized Controlled Trials. Adv. Nutr..

[11] Wallace T.C., Giusti M.M. (2019). Anthocyanins-Nature’s Bold, Beautiful, and Health-Promoting Colors. Foods.

[12] Borges G., Degeneve A., Mullen W., Crozier A. (2010). Identification of flavonoid and phenolic antioxidants in black currants, blueberries, raspberries, red currants, and cranberries. J. Agric. Food Chem..

[13] Xu L., Tian Z., Chen H., Zhao Y., Yang Y. (2021). Anthocyanins, Anthocyanin-Rich Berries, and Cardiovascular Risks: Systematic Review and Meta-Analysis of 44 Randomized Controlled Trials and 15 Prospective Cohort Studies. Front. Nutr..

[14] Wallace T.C., Giusti M.M. (2015). Anthocyanins. Adv. Nutr..

[15] Wallace T.C., Bailey R.L., Blumberg J.B., Burton-Freeman B., Chen C.O., Crowe-White K.M., Drewnowski A., Hooshmand S., Johnson E., Lewis R. (2020). Fruits, vegetables, and health: A comprehensive narrative, umbrella review of the science and recommendations for enhanced public policy to improve intake. Crit. Rev. Food Sci. Nutr..

[16] Vogiatzoglou A., Mulligan A.A., Lentjes M.A., Luben R.N., Spencer J.P., Schroeter H., Khaw K.T., Kuhnle G.G. (2015). Flavonoid intake in European adults (18 to 64 years). PLoS ONE.

[17] Ovaskainen M.L., Torronen R., Koponen J.M., Sinkko H., Hellstrom J., Reinivuo H., Mattila P. (2008). Dietary intake and major food sources of polyphenols in Finnish adults. J. Nutr..

[18] U.S. Department of Agriculture ARS (2022). Flavonoid Intakes from Food and Beverages: Mean Amounts Consumed per Individual, by Gender and Age, What We Eat in America, NHANES 2017–2018. https://www.ars.usda.gov/northeast-area/beltsville-md-bhnrc/beltsville-human-nutrition-research-center/food-surveys-research-group/docs/fndds-flavonoid-database/.

[19] Golovinskaia O., Wang C.K. (2021). Review of Functional and Pharmacological Activities of Berries. Molecules.

[20] Njus D., Kelley P.M., Tu Y.J., Schlegel H.B. (2020). Ascorbic acid: The chemistry underlying its antioxidant properties. Free Radic. Biol. Med..

[21] Si H., Liu D. (2014). Dietary antiaging phytochemicals and mechanisms associated with prolonged survival. J. Nutr. Biochem..

[22] Kalt W., Cassidy A., Howard L.R., Krikorian R., Stull A.J., Tremblay F., Zamora-Ros R. (2020). Recent Research on the Health Benefits of Blueberries and Their Anthocyanins. Adv. Nutr..

[23] Martini D., Marino M., Venturi S., Tucci M., Klimis-Zacas D., Riso P., Porrini M., Del Bo C. (2023). Blueberries and their bioactives in the modulation of oxidative stress, inflammation and cardio/vascular function markers: A systematic review of human intervention studies. J. Nutr. Biochem..

[24] Onali T., Kivimaki A., Mauramo M., Salo T., Korpela R. (2021). Anticancer Effects of Lingonberry and Bilberry on Digestive Tract Cancers. Antioxidants.

[25] Page M.J., McKenzie J.E., Bossuyt P.M., Boutron I., Hoffmann T.C., Mulrow C.D., Shamseer L., Tetzlaff J.M., Akl E.A., Brennan S.E. (2021). The PRISMA 2020 statement: An updated guideline for reporting systematic reviews. BMJ.

[26] Rethlefsen M.L., Kirtley S., Waffenschmidt S., Ayala A.P., Moher D., Page M.J., Koffel J.B., Group P.-S. (2021). PRISMA-S: An extension to the PRISMA Statement for Reporting Literature Searches in Systematic Reviews. Syst. Rev..

[27] Lefebvre C., Glanville J., Briscoe S., Featherstone R., Littlewood A., Marshall C., Metzendorf M.-I., Noel-Storr A., Paynter R., Rader T., Higgins J.P.T., Thomasm J., Chandler J., Cumpston M., Li T., Page M.J., Welch V.A. (2022). Chapter 4: Searching for and Selecting Studies. Cochrane Handbook for Systematic Reviews of Interventions.

[28] Higgins J.P., Altman D.G., Gotzsche P.C., Juni P., Moher D., Oxman A.D., Savovic J., Schulz K.F., Weeks L., Sterne J.A. (2011). The Cochrane Collaboration’s tool for assessing risk of bias in randomised trials. BMJ.

[29] Arevstrom L., Bergh C., Landberg R., Wu H., Rodriguez-Mateos A., Waldenborg M., Magnuson A., Blanc S., Frobert O. (2019). Freeze-dried bilberry (*Vaccinium myrtillus*) dietary supplement improves walking distance and lipids after myocardial infarction: An open-label randomized clinical trial. Nutr. Res..

[30] Asgary S., Soltani R., Mirvakili S., Sarrafzadegan N. (2016). Evaluation of the effect of *Vaccinium arctostaphylos* L. fruit extract on serum inflammatory biomarkers in adult hyperlipidemic patients: A randomized double-blind placebo-controlled clinical trial. Res. Pharm. Sci..

[31] Basu A., Betts N.M., Nguyen A., Newman E.D., Fu D., Lyons T.J. (2014). Freeze-dried strawberries lower serum cholesterol and lipid peroxidation in adults with abdominal adiposity and elevated serum lipids. J. Nutr..

[32] Bowtell J.L., Aboo-Bakkar Z., Conway M.E., Adlam A.R., Fulford J. (2017). Enhanced task-related brain activation and resting perfusion in healthy older adults after chronic blueberry supplementation. Appl. Physiol. Nutr. Metab..

[33] Chan S.W., Chu T.T.W., Choi S.W., Benzie I.F.F., Tomlinson B. (2021). Impact of short-term bilberry supplementation on glycemic control, cardiovascular disease risk factors, and antioxidant status in Chinese patients with type 2 diabetes. Phytother. Res..

[34] Chew B., Mathison B., Kimble L., McKay D., Kaspar K., Khoo C., Chen C.O., Blumberg J. (2019). Chronic consumption of a low calorie, high polyphenol cranberry beverage attenuates inflammation and improves glucoregulation and HDL cholesterol in healthy overweight humans: A randomized controlled trial. Eur. J. Nutr..

[35] Curtis P.J., van der Velpen V., Berends L., Jennings A., Feelisch M., Umpleby A.M., Evans M., Fernandez B.O., Meiss M.S., Minnion M. (2019). Blueberries improve biomarkers of cardiometabolic function in participants with metabolic syndrome-results from a 6-month, double-blind, randomized controlled trial. Am. J. Clin. Nutr..

[36] Davinelli S., Bertoglio J.C., Zarrelli A., Pina R., Scapagnini G. (2015). A Randomized Clinical Trial Evaluating the Efficacy of an Anthocyanin-Maqui Berry Extract (Delphinol(R)) on Oxidative Stress Biomarkers. J. Am. Coll. Nutr..

[37] de Liz S., Cardoso A.L., Copetti C.L.K., Hinnig P.F., Vieira F.G.K., da Silva E.L., Schulz M., Fett R., Micke G.A., Di Pietro P.F. (2020). Acai (*Euterpe oleracea* Mart.) and jucara (*Euterpe edulis* Mart.) juices improved HDL-c levels and antioxidant defense of healthy adults in a 4-week randomized cross-over study. Clin. Nutr..

[38] Ekhlasi G., Shidfar F., Agah S., Merat S., Hosseini A.F. (2015). Effects of Pomegranate and Orange Juice on Antioxidant Status in Non-Alcoholic Fatty Liver Disease Patients: A Randomized Clinical Trial. Int. J. Vitam. Nutr. Res..

[39] Espinosa-Moncada J., Marin-Echeverri C., Galvis-Perez Y., Ciro-Gomez G., Aristizabal J.C., Blesso C.N., Fernandez M.L., Barona-Acevedo J. (2018). Evaluation of Agraz Consumption on Adipocytokines, Inflammation, and Oxidative Stress Markers in Women with Metabolic Syndrome. Nutrients.

[40] Hsia D.S., Zhang D.J., Beyl R.S., Greenway F.L., Khoo C. (2020). Effect of daily consumption of cranberry beverage on insulin sensitivity and modification of cardiovascular risk factors in adults with obesity: A pilot, randomised, placebo-controlled study. Br. J. Nutr..

[41] Hurst R.D., Lyall K.A., Wells R.W., Sawyer G.M., Lomiwes D., Ngametua N., Hurst S.M. (2020). Daily Consumption of an Anthocyanin-Rich Extract Made From New Zealand Blackcurrants for 5 Weeks Supports Exercise Recovery Through the Management of Oxidative Stress and Inflammation: A Randomized Placebo Controlled Pilot Study. Front. Nutr..

[42] Hutchison A.T., Flieller E.B., Dillon K.J., Leverett B.D. (2016). Black Currant Nectar Reduces Muscle Damage and Inflammation Following a Bout of High-Intensity Eccentric Contractions. J. Diet. Suppl..

[43] Johnson S.A., Figueroa A., Navaei N., Wong A., Kalfon R., Ormsbee L.T., Feresin R.G., Elam M.L., Hooshmand S., Payton M.E. (2015). Daily blueberry consumption improves blood pressure and arterial stiffness in postmenopausal women with pre- and stage 1-hypertension: A randomized, double-blind, placebo-controlled clinical trial. J. Acad. Nutr. Diet..

[44] Khan F., Ray S., Craigie A.M., Kennedy G., Hill A., Barton K.L., Broughton J., Belch J.J. (2014). Lowering of oxidative stress improves endothelial function in healthy subjects with habitually low intake of fruit and vegetables: A randomized controlled trial of antioxidant- and polyphenol-rich blackcurrant juice. Free Radic. Biol. Med..

[45] Kim H., Simbo S.Y., Fang C., McAlister L., Roque A., Banerjee N., Talcott S.T., Zhao H., Kreider R.B., Mertens-Talcott S.U. (2018). Acai (*Euterpe oleracea* Mart.) beverage consumption improves biomarkers for inflammation but not glucose- or lipid-metabolism in individuals with metabolic syndrome in a randomized, double-blinded, placebo-controlled clinical trial. Food Funct..

[46] Marin-Echeverri C., Blesso C.N., Fernandez M.L., Galvis-Perez Y., Ciro-Gomez G., Nunez-Rangel V., Aristizabal J.C., Barona-Acevedo J. (2018). Effect of Agraz (*Vaccinium meridionale* Swartz) on High-Density Lipoprotein Function and Inflammation in Women with Metabolic Syndrome. Antioxidants.

[47] McAnulty L.S., Collier S.R., Landram M.J., Whittaker D.S., Isaacs S.E., Klemka J.M., Cheek S.L., Arms J.C., McAnulty S.R. (2014). Six weeks daily ingestion of whole blueberry powder increases natural killer cell counts and reduces arterial stiffness in sedentary males and females. Nutr. Res..

[48] Mohammed M.B.R., Razzaq B.A., Al-Naqqash M., Jasim S.Y. (2016). Effects of Cranberry-PACs against Urinary Problems associated with Radiotherapy in Iraqi Patients with Bladder Carcinoma. Int. J. Pharm. Sci. Rev. Res..

[49] Nilsson A., Salo I., Plaza M., Bjorck I. (2017). Effects of a mixed berry beverage on cognitive functions and cardiometabolic risk markers; A randomized cross-over study in healthy older adults. PLoS ONE.

[50] Paquette M., Medina Larque A.S., Weisnagel S.J., Desjardins Y., Marois J., Pilon G., Dudonne S., Marette A., Jacques H. (2017). Strawberry and cranberry polyphenols improve insulin sensitivity in insulin-resistant, non-diabetic adults: A parallel, double-blind, controlled and randomised clinical trial. Br. J. Nutr..

[51] Petrovic S., Arsic A., Glibetic M., Cikiriz N., Jakovljevic V., Vucic V. (2016). The effects of polyphenol-rich chokeberry juice on fatty acid profiles and lipid peroxidation of active handball players: Results from a randomized, double-blind, placebo-controlled study. Can. J. Physiol. Pharmacol..

[52] Riso P., Klimis-Zacas D., Del Bo C., Martini D., Campolo J., Vendrame S., Moller P., Loft S., De Maria R., Porrini M. (2013). Effect of a wild blueberry (*Vaccinium angustifolium*) drink intervention on markers of oxidative stress, inflammation and endothelial function in humans with cardiovascular risk factors. Eur. J. Nutr..

[53] Sangild J., Faldborg A., Schousboe C., Fedder M.D.K., Christensen L.P., Lausdahl A.K., Arnspang E.C., Gregersen S., Jakobsen H.B., Knudsen U.B. (2023). Effects of Chokeberries (*Aronia* spp.) on Cytoprotective and Cardiometabolic Markers and Semen Quality in 109 Mildly Hypercholesterolemic Danish Men: A Prospective, Double-Blinded, Randomized, Crossover Trial. J. Clin. Med..

[54] Stote K.S., Sweeney M.I., Kean T., Baer D.J., Novotny J.A., Shakerley N.L., Chandrasekaran A., Carrico P.M., Melendez J.A., Gottschall-Pass K.T. (2017). The effects of 100% wild blueberry (*Vaccinium angustifolium*) juice consumption on cardiometablic biomarkers: A randomized, placebo-controlled, crossover trial in adults with increased risk for type 2 diabetes. BMC Nutr..

[55] Terrazas S., Galan B.S.M., De Carvalho F.G., Venancio V.P., Antunes L.M.G., Papoti M., Toro M.J.U., da Costa I.F., de Freitas E.C. (2020). Acai pulp supplementation as a nutritional strategy to prevent oxidative damage, improve oxidative status, and modulate blood lactate of male cyclists. Eur. J. Nutr..

[56] Xie L., Vance T., Kim B., Lee S.G., Caceres C., Wang Y., Hubert P.A., Lee J.Y., Chun O.K., Bolling B.W. (2017). Aronia berry polyphenol consumption reduces plasma total and low-density lipoprotein cholesterol in former smokers without lowering biomarkers of inflammation and oxidative stress: A randomized controlled trial. Nutr. Res..

[57] Turner N.D., Lupton J.R. (2021). Dietary Fiber. Adv. Nutr..

[58] Baptista S.L., Copetti C.L.K., Cardoso A.L., Di Pietro P.F. (2021). Biological activities of acai (*Euterpe oleracea* Mart.) and jucara (*Euterpe edulis* Mart.) intake in humans: An integrative review of clinical trials. Nutr. Rev..

[59] Redan B.W., Buhman K.K., Novotny J.A., Ferruzzi M.G. (2016). Altered Transport and Metabolism of Phenolic Compounds in Obesity and Diabetes: Implications for Functional Food Development and Assessment. Adv. Nutr..

[60] Galano J.M., Lee Y.Y., Durand T., Lee J.C. (2015). Special Issue on “Analytical Methods for Oxidized Biomolecules and Antioxidants” The use of isoprostanoids as biomarkers of oxidative damage, and their role in human dietary intervention studies. Free Radic. Res..

[61] Klawitter J., Haschke M., Shokati T., Klawitter J., Christians U. (2011). Quantification of 15-F2t-isoprostane in human plasma and urine: Results from enzyme-linked immunoassay and liquid chromatography/tandem mass spectrometry cannot be compared. Rapid Commun. Mass Spectrom..

[62] Milne G.L. (2017). Classifying oxidative stress by F_2_-Isoprostane levels in human disease: The re-imagining of a biomarker. Redox Biol..

[63] van ’t Erve T.J., Kadiiska M.B., London S.J., Mason R.P. (2017). Classifying oxidative stress by F_2_-isoprostane levels across human diseases: A meta-analysis. Redox Biol..

[64] Aluganti Narasimhulu C., Parthasarathy S. (2022). Preparation of LDL, Oxidation, Methods of Detection, and Applications in Atherosclerosis Research. Methods Mol. Biol..

[65] Munteanu I.G., Apetrei C. (2021). Analytical Methods Used in Determining Antioxidant Activity: A Review. Int. J. Mol. Sci..

[66] Papadea P., Skipitari M., Kalaitzopoulou E., Varemmenou A., Spiliopoulou M., Papasotiriou M., Papachristou E., Goumenos D., Onoufriou A., Rosmaraki E. (2022). Methods on LDL particle isolation, characterization, and component fractionation for the development of novel specific oxidized LDL status markers for atherosclerotic disease risk assessment. Front. Med..

[67] Candel M., van Breukelen G.J.P. (2023). Best (but oft forgotten) practices: Efficient sample sizes for commonly used trial designs. Am. J. Clin. Nutr..

[68] Cerda B., Periago P., Espin J.C., Tomas-Barberan F.A. (2005). Identification of urolithin a as a metabolite produced by human colon microflora from ellagic acid and related compounds. J. Agric. Food Chem..

[69] Espin J.C., Gonzalez-Sarrias A., Tomas-Barberan F.A. (2017). The gut microbiota: A key factor in the therapeutic effects of (poly)phenols. Biochem. Pharmacol..

[70] Luca S.V., Macovei I., Bujor A., Miron A., Skalicka-Wozniak K., Aprotosoaie A.C., Trifan A. (2020). Bioactivity of dietary polyphenols: The role of metabolites. Crit. Rev. Food Sci. Nutr..

[71] van Duynhoven J., Vaughan E.E., Jacobs D.M., Kemperman R.A., van Velzen E.J., Gross G., Roger L.C., Possemiers S., Smilde A.K., Dore J. (2011). Metabolic fate of polyphenols in the human superorganism. Proc. Natl. Acad. Sci. USA.

[72] Boto-Ordonez M., Urpi-Sarda M., Queipo-Ortuno M.I., Tulipani S., Tinahones F.J., Andres-Lacueva C. (2014). High levels of Bifidobacteria are associated with increased levels of anthocyanin microbial metabolites: A randomized clinical trial. Food Funct..

[73] Espley R.V., Butts C.A., Laing W.A., Martell S., Smith H., McGhie T.K., Zhang J., Paturi G., Hedderley D., Bovy A. (2014). Dietary flavonoids from modified apple reduce inflammation markers and modulate gut microbiota in mice. J. Nutr..

[74] Igwe E.O., Charlton K.E., Probst Y.C., Kent K., Netzel M.E. (2019). A systematic literature review of the effect of anthocyanins on gut microbiota populations. J. Hum. Nutr. Diet..

[75] Jennings A., Koch M., Bang C., Franke A., Lieb W., Cassidy A. (2021). Microbial Diversity and Abundance of Parabacteroides Mediate the Associations Between Higher Intake of Flavonoid-Rich Foods and Lower Blood Pressure. Hypertension.

[76] Sweeney M., Burns G., Sturgeon N., Mears K., Stote K., Blanton C. (2022). The Effects of Berry Polyphenols on the Gut Microbiota and Blood Pressure: A Systematic Review of Randomized Clinical Trials in Humans. Nutrients.

[77] Brunelli E., Domanico F., La Russa D., Pellegrino D. (2014). Sex differences in oxidative stress biomarkers. Curr. Drug Targets.

[78] Rosner B. (2016). Fundamentals of Biostatistics.

[79] Higgins T.J., Chandler J., Cumpston M., Li T., Page M.J., Welch V.A. (2021). Cochrane Handbook for Systematic Reviews of Interventions.

